# Challenges in Nurses' Use of Behaviour Change Techniques in Chronic Pain Management

**DOI:** 10.1155/prm/4405696

**Published:** 2025-08-07

**Authors:** Jasmine Heath Hearn, Areeba Rafiq, John Greenwood, Jordan Wilkey, Faith Johnson, Christopher McCarthy

**Affiliations:** ^1^School of Psychology, Manchester Metropolitan University, Manchester, UK; ^2^Connect Health, Newcastle Upon Tyne, UK; ^3^Manchester Movement Unit, Manchester Metropolitan University, Manchester, UK

**Keywords:** behaviour change, chronic pain, implementation science, pain management, qualitative

## Abstract

**Objective:** Patient adherence to treatment recommendations is less than optimal within chronic pain management. Behaviour change techniques (BCTs) and frameworks can be used to maximise engagement with desired behaviours but are also underused. This study sought the perceptions of nurses to explore the perceived barriers and facilitators to utilising BCTs in clinical practice in chronic pain settings.

**Methods:** Eight qualified nurses participated in semi-structured interviews. Reflexive thematic analysis was conducted to understand barriers and facilitators to the use of BCTs in practice.

**Results:** Three themes were identified (1) behaviour change embedded in current practice, (2) complexities in chronic pain as barriers in implementing behaviour change and (3) from experience to expertise: training and supervision needs. Findings suggest that nurses engage in some BCTs (17 were discussed across all interviews), without explicit knowledge of specific BCTs and how to use them. The use of BCTs is restricted by patients' medical complexities, including mental health comorbidities, unhelpful biomedical beliefs about pain and opioid reliance. Furthermore, the opportunity to effectively utilise BCTs is impeded by a lack of training and clinical supervision.

**Conclusions:** Improving nurses' capabilities by enhancing BCT training and clinical supervision is required. Furthermore, organisational change is recommended to create the opportunity for nurses to effectively utilise BCTs. Specifically, organisations should devote necessary resources, backed by effective implementation strategies, to enhance such engagement.

**Summary**

• What does this paper contribute to the wider global clinical community?
◦ Nurses are vital within chronic pain management. They recognise the utility of using behaviour change approaches to improve chronic pain management.
◦ However, nurses feel limited in their use of behaviour change techniques and approaches by a lack of training and clinical supervision, and work is required to address this skills gap.
◦ Embedding training in behaviour change across the training pathway, and in speciality training, will be a beneficial adjustment to equip nurses to more effectively facilitate behaviour change.

## 1. Introduction

Chronic pain is a significant global issue, affecting approximately 43% of the UK population [1]. It imposes a heavy psychological burden and a high incidence of comorbidities, significantly impacting social functioning [2]. Chronic pain places considerable economic strain, with back pain estimated to cost the UK economy £12 billion annually [3] and $8.15 billion globally [4]. Adherence to chronic pain management is crucial to mitigate its effects on both individuals and society.

Adherence, “the extent to which a person's behaviour corresponds with agreed recommendations from a healthcare provider” [5], is a crucial predictor of treatment efficacy in chronic pain [6]. However, nonadherence to treatment and lifestyle recommendations is prevalent [7]. Nonadherence rates to prescriptions range from 8% to 62% [8], and only 8% of people with chronic low back pain fully adhere to walking recommendations [9]. Nonadherence is therefore a significant challenge for clinicians, patients and the healthcare system.

The behaviour change technique (BCT) taxonomy [10], a framework of 93 BCTs (e.g., goal setting and habit formation), can be applied by health professionals to facilitate behaviour change and is evidenced to improve patient outcomes. With nurses playing a fundamental role in managing chronic pain (e.g., assessment, advice, monitoring and evaluation of pain [11, 12]), their versatility and frequent patient contact makes themwell-suited to utilise BCTs to improve patient adherence and health outcomes. Indeed, nurse-led interventions (e.g., education programs) can increase patient knowledge and perceived control of pain [13].

Despite their frontline positions, research into nurses' use of BCTs in chronic pain care is limited. Frameworks for improving health behaviours through making every contact count (MECC) exist [14], using the three A's (ask, advise and assist) and feedback, responsibility, advice, menu of options, empathy and self-efficacy (FRAMES) to empower patients to adopt healthy behaviours. However, none of these frameworks explicitly reference BCTs to promote behavioural change, and evidence suggests that nurses rely on information provision and reinforcing positive behaviours rather than addressing issues like motivation [15], negative beliefs [16] or goal setting [17], which may be more effective in promoting behaviour change.

Nurses desire further training in navigating behaviour change conversations with patients in weight management [18]. This is also the case in diabetes care, where nurses are reluctant to engage in behaviour change discussions with patients [19], and nurses infrequently use effective techniques to support behaviour change for self-management [20]. For chronic pain, there is a lack of evidence surrounding nurses' understandings of behaviour change, and barriers and facilitators to using BCTs to promote behaviour change and adherence to recommendations. This study aimed to address this gap to understand if/how nurses use BCTs in chronic pain care, and barriers and facilitators to implementing them.

## 2. Methods

### 2.1. Design

A qualitative, semi-structured interview study was utilised to explore perceived barriers and facilitators to use of BCT and to identify areas for change [21].

### 2.2. Participants

Convenience sampling was applied, with advertisements shared via UK universities and with graduates on postgraduate training pathways for nurses, and on social media (X and LinkedIn) asking participants meeting the inclusion criteria to volunteer for the study. The sample size of eight participants was determined based on the scope of the research and participant requirements [22]. Inclusion criteria were as follows: practicing nurses over 18 years old, registered with the Health and Care Professions Council, and at least 12 months postqualifying experience.

### 2.3. Procedure

Gatekeepers (university course leaders) assisted with recruitment by circulating an interview invitation via email to their course graduates. Eligible participants self-identified by contacting the research team via email to express interest. They were provided with a participation information sheet detailing the study in full. Participants were asked to complete a short demographics questionnaire prior to the interview. All interviews were conducted by JG using the Microsoft Teams recording and transcription feature. At the start of each interview, audio consent was recorded.

Semi-structured interviews were conducted, allowing the researcher flexibility to pursue topics as they arose, obtaining rich data and subjective insights from participants [23]. An interview schedule was developed based on broad reading of the evidence base and discussion among the research team. This was piloted with a lecturer in nursing to ensure that questions were appropriate and provided sufficient opportunity for participants to discuss their knowledge and skills. Open questions were utilised during the interview starting with a broad opening aimed at putting participants at ease. Further questioning techniques were applied throughout, including examples, prompting and summary questions. Probing questions were also used to seek clarity and detail and enhance data credibility [24]. The full interview schedule is displayed in Table 1.

### 2.4. Data Analysis

Data were analysed by JHH and AR using reflexive thematic analysis, a theoretically flexible method for identifying, analysing and interpreting patterns of meaning within qualitative data [22]. The analytical process followed six distinct steps as set out by Braun and Clarke [22]; familiarisation; coding; generating initial themes; developing and reviewing themes; refining, defining and naming themes; and writing up. Familiarisation involved immersion with the data by watching the video interviews, checking the accuracy of transcripts and re-reading multiple times while logging initial considerations and any similarities/differences between transcripts. When transcribing, data were also cleaned by removing repetition, hesitation and adding punctuation as described by Braun and Clarke [22]. The full dataset was reviewed, and code labels were attached to segments of the data which were relevant to the research question.

A semantic approach was applied to coding, to present the data as communicated by nurses, thereby explicitly understanding their views of barriers and facilitators to the use of BCTs. This was deemed appropriate as the research was focused on the specific topic of BCTs and the practical, everyday use of these techniques rather than seeking to identify hidden meaning, emotion or underlying ideologies which may have been contributing to the data. When generating initial themes, potentially connected codes were clustered into candidate themes to explore their meaning in relation to the research question. An inductive approach was taken to initial theme identification as the research was exploratory. Following this, themes were developed and reviewed by the research team. During this stage, all code labels were revisited, ensuring that they were informed by meaningful data and presented a coherent pattern with relevance to both the proposed theme and research question. When this process was complete, the final themes were defined and named to reflect the data within them.

### 2.5. Methods to Ensure Rigour

To ensure rigour, dependability was demonstrated by providing transparency and details regarding the methods of data collection, analysis and interpretation. The participants' voice is represented alongside detailed contextual information, and the research team undertook reflexivity throughout to remain transparent regarding their own subjectivity and potential bias which may influence the research process [25]. It should be noted that the research team included students, a qualified occupational therapist, a qualified consultant physiotherapist and an academic psychologist, all specialising in chronic pain management, and were therefore able to relate to nurses' experiences. All authors are interested in the use of BCTs in clinical practice and therefore have specialist knowledge of behaviour change frameworks, theories and techniques. This influenced the decision to pursue this research and may also have informed underlying assumptions about the benefits of BCTs in nursing practice. To mitigate this, semantic coding, multiple reviews of data extracts and regular team discussions regarding the findings ensured that the perspectives of the participants were provided, rather than those of the researchers.

### 2.6. Ethical Considerations

Ethical approval was granted by the Manchester Metropolitan University Research Ethics Committee (Ref.: 53858). All participants were provided with detailed study information and provided informed consent to participate. Upon completion of interview transcription, all recordings were destroyed and personal data (such as names and places discussed in interviews) were replaced with pseudonyms. Data were anonymised using a unique pseudonym, kept confidential and only used for the stated aims of the research.

### 2.7. Patient and Public Involvement

Two qualified nurses who were also involved in training undergraduate nurses were involved in PPI discussions to support the study design, data collection methods and interpretation of findings. They were directly involved in determining study aims, developing the interview questions, piloting the interview and reviewing the findings (for example, PPI discussions helped us understand the complexities of nursing practice in chronic pain settings and the competing demands nurses face when supporting patients). Their feedback has been integrated into the final report.

## 3. Results

The final sample (N = 8, F = 7 and M = 1) consisted of nurses with an age range of 29–53 years (*M* = 38.75). Participants predominantly worked in specialist clinics and hospitals, and two had received formal training in behaviour change-related techniques, including motivational interviewing and cognitive behavioural therapy (which were completed as additional training after their qualification as nurses). A detailed summary of participant demographics is shown in Table 2.

Three themes were identified as follows: (1) behaviour change embedded in current practice, (2) complexities in chronic pain as barriers in implementing behaviour change and (3) from experience to expertise: training and supervision needs. Together, these themes provide insights into the barriers and facilitators perceived by nurses to the use of BCTs to improve patient outcomes.

### 3.1. Behaviour Change Embedded in Current Practice

Participants reflected on their current practice, encompassing a widely held belief: one of their primary goals in facilitating patient behaviour change is to empower patients to have agency for their own health. Nurses organically achieved this by fostering a supportive environment where patients can make informed decisions about their care:

It's all about what they want and their own kind of journey with it … they should want to do it for themselves, not because I've told them or I've offered them something. (Gemma)

Nurses view patients as partners, respecting their values, preferences and individual goals and used these to enhance intrinsic motivation and self-efficacy in patients, often using behaviour change principles naturally. Seventeen techniques were discussed across all interviews, with the most common being goal setting, graded tasks, social support, self-monitoring of outcomes, information about health consequences, framing/reframing and problem-solving (see Table 3 for a detailed list and definitions). These were discussed as general strategies used by participants, rather than being specifically chosen because of their knowledge of BCTs. Setting value-based goals was frequently highlighted as a primary task during appointments, emphasizing the importance of patient-decided goals and problem-solving techniques which help patients identify barriers preventing behaviour change:

Often the root of low motivation was the goal not being personal to the patient, which nurses were able to pick up on with ease. Allowing patients to make decisions about their treatment increases their commitment and ownership of their health journey by making it personal. Graded tasks, increasing in difficulty, were instrumental in achieving these goals. This approach ensured that patients are able to achieve small goals and served as a motivating factor for patients to continue engaging while monitoring positive outcomes:

Social support (unspecified, practical and emotional) during follow-up appointments from staff and praise from close ones enhanced patient motivation. Encouraging patients to share their behavioural outcomes with family and friends helped realise their achievements and thus increase motivation. However, nurses raised concerns about not providing excessive praise to their patients to avoid a paternalistic approach that might be viewed as patronising:

[I don't want to] praise them. Because what I don't want is the reliance on me that they're looking at me to give them answers and praising them can sometimes cause that. (Fran)

Nurses reflected on their awareness of behaviour change and its necessity in all areas of nursing but were aware that particular areas of nursing, such as mental health, were prioritised in training. Consequently, those nurses who naturally utilize BCTs rely on their clinical experience, rather than on formal training received in their current roles as specialist nurses in chronic pain management:

Some of these behaviour change techniques, people are using them, but they don't necessarily know that they are behaviour change. I think it's just something that I've learned over the years in nursing and obviously just not been told that's what I'm doing … they should incorporate it into the newly qualified nurses so they're more aware of it. (Lindsey)

This theme demonstrates how nurses work in a behaviourally informed manner, even without formal training/awareness of BCTs. They are able to foster a supportive environment, respecting values and preferences, and ensure that treatment plans align with individual goals, maintain motivation and adherence and encourage patients to make informed decisions about their care.

### 3.2. Complexities in Chronic Pain as Barriers in Implementing Behaviour Change

A key element of the interviews was the medical, psychological and social complexities that patients with chronic pain contend with, which can act as barriers to engaging in desired target behaviours and pose challenges for nurses in implementing behaviour change strategies. The most common challenges were patients' fear and biomedical understandings of chronic pain:

A lot of the barriers is around fear. They believe that the medication is the be all end all. They feel that it's working even though they tell me that it's [pain] intermittent, some days are better than others, which to me indicates that it's probably not helping. (Fran)

There's a lot of challenges in the respects of, I guess they don't want to change and all they're scared to change because they're in pain or because they're not used to it, or because they think that if they do change, it might hurt them. (Margaret)

Unhelpful beliefs about pain and the potential impact of behaviours on pain have to be addressed first before any behaviour change intervention can be implemented. Nurses, as the often first point of contact for patients, are at the forefront of these challenges, playing a pivotal role in helping patients overcome them and often priority needs to be placed on undoing unhelpful beliefs/understandings of pain and expectations of care:

Quite often you do get the [response from patients], “well, I want the pain to go away” and then you have to start explaining like “no, no, it's not going to go away completely”. (Gemma)

Completely understandably, some people would like a tablet to take, or some people would like something to be done to them, maybe an injection to be given to them and then the pain will just go away and a conversation around that … can be really useful for people. It can be a very hard conversation, but I think it's an absolutely essential thing to do. (James)

Co-occurring pain medication dependence alongside chronic pain may induce patient fear when considering changing from a medicalised approach to a behaviour-focused approach. Open and honest conversations are crucial to navigate this transition and enable patients to engage in behavioural change. However, challenges in the medical system may complicate this, with nurses reflecting on examples of prescribed medications being left as repeat prescriptions, going unchecked and leading to dependency, as well as the time constraints of GP consultations which can leave patients with a limited understanding of their situation and the relevance of behaviour change to their pain:

The knowledge is the key thing because quite often the GPs will have prescribed it, or it may have been that the patient was in hospital, say, had joint replacement surgery and was started on co-codamol and because the GP's limited for time with his patients, that just gets put on repeat prescription. Before you know it, the patient's dependent on it and then it's a very tricky situation to try and get them off it even though everything is healed. (Fran)

Again, when at the forefront of patient care, more pressing issues such as dependence must take precedence before approaching other aspects of care, such as addressing patient knowledge and discussions around behaviour change. This also extends to mental health concerns and communication, which are also priority areas of focus for nurses:

They may be dealing with a [mental health] crisis at the moment, and it can be really difficult sometimes to get the patients to engage with our service. (Nia)

I guess we might do some role modelling … That's always good, because communication is normally a significant problem for people with long-term pain. They don't want to burden other people. They don't want to be a bother … They're super nice people and they don't want to be a problem, so communication around pain is really difficult. (James)

This theme explored obstacles hindering patient engagement in, and adherence to, behaviour change, with unhelpful biomedical beliefs, fear and medication dependency emerging as key challenges that require to be addressed before any focus on behaviour change. Nurses strive to address these barriers sensitively and as priority but recognise that these may prevent them from reaching appropriate and effective behaviour change to support patients to manage their pain.

### 3.3. From Experience to Expertise: Training and Supervision Needs

Nurses reflected on the lack of formal awareness of behaviour change and BCTs within their discipline, whilst recognising the unconscious yet prevalent use of these techniques in their practice. They recognised where behaviour change training/knowledge is prioritised in nursing training/practice:

I find that they just target mental health nurses with behaviour changes … I think behaviour change applies to everyone now. (Lindsey)

The prioritisation of behaviour changes within mental health nursing means that other nursing specialities lose out on behaviour change training, even where it is highly relevant. Where participants were aware of behaviour change and BCTs, this awareness was gleaned from learning “on-the-job” (though this was uncommon among participants):

No, not [trained in behaviour change] at all. It's just things I've picked up along the way, really. (Nia)

Nurses were passionate about improving outcomes for patients and had attempted to integrate behaviour change approaches into their practice. However, without formal training, nurses felt unprepared and unsupported to implement such approaches:

You need more in-depth training to fully understand what it actually is and how to implement it in practice … I've had some information on it and I've made notes, but I'll be honest, I haven't really practiced it because I didn't fully understand what it is and the best ways to go about it. (Fran)

Despite their on-the-job learning (and some prior training in ACT and motivational interviewing for Fran), participants agreed that formal training and supervision were required. Ongoing training and supervision and opportunities to reflect on their practice would increase their confidence and proficiency in applying behaviour change approaches, particularly important given the need for nurses to quickly devise solutions to changing patient situations:

Training days where you can ask those questions or give examples of maybe more difficult conversations or situations you've had with patients where maybe from the limited stuff that I know about or that I've practiced where that actually doesn't feel like it's worked. (Gemma)

Participants explained that formal training, shadowing and supervision would allow them to practice behaviour change approaches and increase their confidence, knowing that there was a safe and supportive environment for skill development. This was particularly important given that participants were wary of using skills they did not feel confident navigating:

Really, education and role play and use with appropriate supervision. I think to reduce the fear involved in using it because as a clinician, any technique that you're going to be using with a patient that potentially could upset them needs supervision. No one wants to hurt patients. (James)

This theme highlights that despite a lack of awareness of its theoretical basis and limited formal training in behaviour change, participants were motivated to embed behaviour change approaches in their work. However, they emphasised the need for supervision and ongoing training to effectively and safely apply BCTs in patient care.

## 4. Discussion

This study aimed to understand the ways in which nurses use BCTs in clinical settings and barriers and facilitators to implementing them. Eight participants completed semi-structured interviews, with three themes identified: (1) behaviour change embedded in current practice, (2) complexities in chronic pain as barriers in implementing behaviour change and (3) from experience to expertise: training and supervision needs.

Nurses organically embedded some behaviour change principles in their practice by fostering a supportive environment where patients can make informed decisions about their care, often using formal BCTs, such as goal setting, graded tasks, self-monitoring of outcomes, information about health consequences and reframing, without explicitly recognising these as BCTs. This enabled nurses to support their patients to develop self-efficacy to take ownership of their health. However, nurses also acknowledged that they were not confident in their use of BCTs and would benefit from training and supervision to clarify and enhance their skills. Though this has been previously identified as a priority area for enhancing the nursing workforce skills [26], the current research has identified that this skill gap remains and should be prioritised to enhance chronic pain care.

It is evident that the factors influencing nurses' use of and engagement in conversations surrounding health behaviour change for people with chronic pain are complex. Nurses reported having to navigate complex cases in their work, including mental health concerns and medication reliance, which must be prioritised before they engage in behaviour change discussions. This is especially important since people with chronic pain are likely to present with psychiatric comorbidities; research shows that 18%–32% of chronic low back pain patients are found to have major depression during the course of their treatment [27].

Nurses reflected on unhelpful beliefs held by patients surrounding the utility of pain medication, seeing them as the more effective pain relievers, despite evidence and current policy efforts to the contrary [28]. Nurses have to negotiate medication use, which can strain the patient–provider relationship, but is necessary to convince patients that medication should not be solely relied upon in preference to other pain management approaches, and discussions surrounding health behaviours at sensitive time periods may impede this goal [29]. The present research highlights that nurses need support to effectively navigate difficult conversations with patients to support this endeavour.

Whilst nurses reflected on their spontaneous use of behaviour change principles in their practice, this was a result of learning through experience, and their use of BCTs was neither wholly consistent nor a product of BCT expertise. It is important to provide formal training to ensure the effective and safe delivery of BCTs for each patient. Prior work investigating nurses' use of BCTs in a trial to promote physical activity has found that nurses find training in BCT delivery enhanced the quality and delivery of advice and support they provided within routine consultations [30]. However, the present work indicates a need for training to be embedded early in the training pathway, such as undergraduate and postgraduate curricula.

For qualified nurses, the importance of effective clinical supervision is a notable requirement. Participants suggested that they may avoid using potentially useful BCTs unless they had supportive clinical supervision. As such, the provision of psychology-led clinical supervision in BCTs may be an advantageous adjunct to support nurses to develop skills and confidence. This has also been identified in other recent research as a priority area for physiotherapists (Greenwood et al., in review), demonstrating a need for service-wide support for the development of skills, knowledge and confidence in using behaviour change approaches in chronic pain settings.

### 4.1. Implications and Future Research

The findings suggest a range of potential interventions that influence capability (BCT training and skill development), opportunity (addressing structural/organisation barriers to BCT implementation, such as providing more time for behaviour change conversations with patients) and motivation (e.g., improving confidence in BCT use), in line with the COM-B model of behaviour change [31]. The COM-B model is widely used to identify and understand determinants of behaviour through capabilities (capacity to engage in behaviour), opportunity (environmental factors that influence behaviours) and motivation (the willingness to change), factors known to influence an individual's capacity to adopt new behaviours. This has the advantage of enabling the development of theoretically driven individually tailored interventions to support and empower nurses to develop their skills and implement BCTs in practice. This study provides the foundation for developing theory and evidence-based interventions to improve nursing practice and outcomes for their patients.

### 4.2. Strengths and Limitations

This study was novel in its specific focus on BCTs and nurses' perceptions of the barriers and facilitators to their use. It has provided new insights, and a foundation for consideration of interventions aimed at improving nurses' knowledge and confidence of BCTs. However, there are limitations to consider; the content of undergraduate and CPD training available and undertaken by participants was not verified. As such, any recommendations regarding the development of tailored training in behaviour change must be considered in the context of what is currently provided within educational programmes. This study also focused on a small sample of nurses working within chronic pain contexts, and as such, the results may not be more broadly transferable or applicable to nurses working in other contexts. Future research should seek to clarify the extent to which the perceptions and challenges identified in this study are shared amongst all nursing specialisms to establish the extent of the skill gap. Further research may account for such limitations, enabling results to be more transferrable and allowing exploration of best practices in nursing education.

## 5. Conclusion

Frequently the co-ordinating axis of interventions for health, nurses are vital to the improvement of population health. They strive for development of their nursing role through skill development, which can increase confidence and job satisfaction, particularly where skills developed for a specific intervention can be utilised with other patients [26]. The present study demonstrated that where nurses are untrained in behaviour change approaches, they tend to avoid using such strategies due to a lack of confidence. As such, embedding training in behaviour change across the training pathway, and in speciality training, will be a beneficial adjustment to equip nurses to more effectively facilitate behaviour change and promote positive patient outcomes.

## Figures and Tables

**Table 1:** Interview schedule.

1. Are you able to talk me through a typical patient appointment?
2. What are some of the challenges you experience when working with patients?
3. What are some common psychosocial factors you encounter during your assessment and treatment planning, and how might you mitigate these? a. Do you have any specific examples of strategies, techniques, questions or prompts that you use?
4. Tell me how you would help patients overcome barriers to adherence with treatment plans? a. What are some examples of strategies, techniques, questions or prompts that you use?
5. What is your understanding of behaviour change techniques (BCTS)? For example, goal setting, social support, reward, habit formation, self-monitoring, reducing negative emotions and framing/reframing?
6. How confident do you feel applying BCTs, and could you provide any examples you use in clinical practice?
7. Are there any specific barriers that restrict your use of BCTs with patients? For example, confidence, time and training?
8. What, if anything, could be done to equip clinicians with the skills and confidence to use BCTs regularly during appointments?
9. Please consider this case study and how you would respond: An elderly patient attends a second appointment with you and is reporting low levels of motivation and self-confidence to engage with their treatment plan. This is resulting in a lack of adherence and clinical improvements. How might you work with them to improve their adherence?
10. In conclusion, how would you sum up your awareness and use of BCTs within clinical appointments?

**Table 2:** Participant demographics.

Pseudonym and sex (F = 7, M = 1)	Age (M = 38.8 years)	Setting of practice (clinics = 75%, hospital = 25%, home working = 12.5%)	Years of clinical experience (3 years = 25%, 4 or more years = 75%)	Received formal training in behaviour change (Y = 25%, N = 75%)
Lindsey (F)	40	Clinics	4 or more	No
Fran (F)	29	Clinics	3	Yes-ACT therapy, motivational interviewing
Margaret (F)	53	Home working and clinics	3	No
Gemma (F)	29	Clinics	4 or more	No
Imani (F)	33	Hospital	4 or more	No
James (M)	48	Clinics	4 or more	Yes—2 years of high-intensity CBT training with NHS
Alexandra (F)	43	Clinics	4 or more	No
Nia (F)	35	Hospital	4 or more	No

**Table 3:** Behaviour change techniques discussed in interviews and corresponding definitions according to BCT taxonomy v1.

Behaviour change techniques discussed in interviews	Definition (according to BCT taxonomy v1)
1.1. Goal setting (behaviour)	Set or agree on a goal defined in terms of the behaviour to be achieved
1.2. Problem-solving	Analyse, or prompt the person to analyse, factors influencing the behaviour and generate or select strategies that include overcoming barriers and/or increasing facilitators
1.3. Goal setting (outcome)	Set or agree on a goal defined in terms of a positive outcome of wanted behaviour
1.4. Action planning	Prompt detailed planning of performance of the behaviour (must include at least one of context, frequency, duration and intensity). Context may be environmental (physical or social) or internal (physical, emotional or cognitive)
1.5. Review behaviour goals	Review behaviour goal(s) jointly with the person and consider modifying goal(s) or behaviour change strategy in light of achievement. This may lead to resetting the same goal, a small change in that goal or setting a new goal instead of (or in addition to) the first, or no change
2.4. Self-monitoring of outcomes of behaviour	Establish a method for the person to monitor and record the outcome(s) of their behaviour as part of a behaviour change strategy
3.1. Social support (unspecified)	Advise on, arrange, or provide social support (e.g., from friends, relatives, colleagues, ‘buddies' or staff) or noncontingent praise or reward for performance of the behaviour. It includes encouragement and counselling but only when it is directed at the behaviour
3.2. Social support (practical)	Advise on, arrange, or provide practical help (e.g., from friends, relatives, colleagues, ‘buddies' or staff) for performance of the behaviour
3.3. Social support (emotional)	Advise on, arrange, or provide emotional social support (e.g., from friends, relatives, colleagues, ‘buddies' or staff) for performance of the behaviour
4.1. Instruction on how to perform a behaviour	Advise or agree on how to perform the behaviour
5.1. Information about health consequences	Provide information (e.g., written, verbal and visual) about health consequences of performing the behaviour
6.1. Demonstration of the behaviour	Provide an observable sample of the performance of the behaviour, directly in person, or indirectly, e.g., via film, pictures, for the person to aspire to or imitate
8.1. Behavioural practice/rehearsal	Prompt practice or rehearsal of the performance of the behaviour one or more times in a context or at a time when the performance may not be necessary, in order to increase habit and skill
8.3. Habit formation	Prompt rehearsal and repetition of the behaviour in the same context repeatedly so that the context elicits the behaviour
8.7. Graded tasks	Set easy-to-perform tasks, making them increasingly difficult, but achievable, until behaviour is performed
11.2. Reduce negative emotions	Advise on ways of reducing negative emotions to facilitate performance of the behaviour
13.2. Framing/reframing	Suggest the deliberate adoption of a perspective or new perspective on behaviour (e.g., its purpose) in order to change cognitions or emotions about performing the behaviour

## Data Availability

Data are available upon reasonable request from the corresponding author.
